# Supplementary figures and images for: Ipsilateral internal carotid artery web and acute ischemic stroke: A cohort study, systematic review and meta-analysis
Source: PLoS One. 2021 Sep 17;16(9):e0257697. doi: 10.1371/journal.pone.0257697 (PMC8448368; doi:10.1371/journal.pone.0257697)

**Supplementary Results**


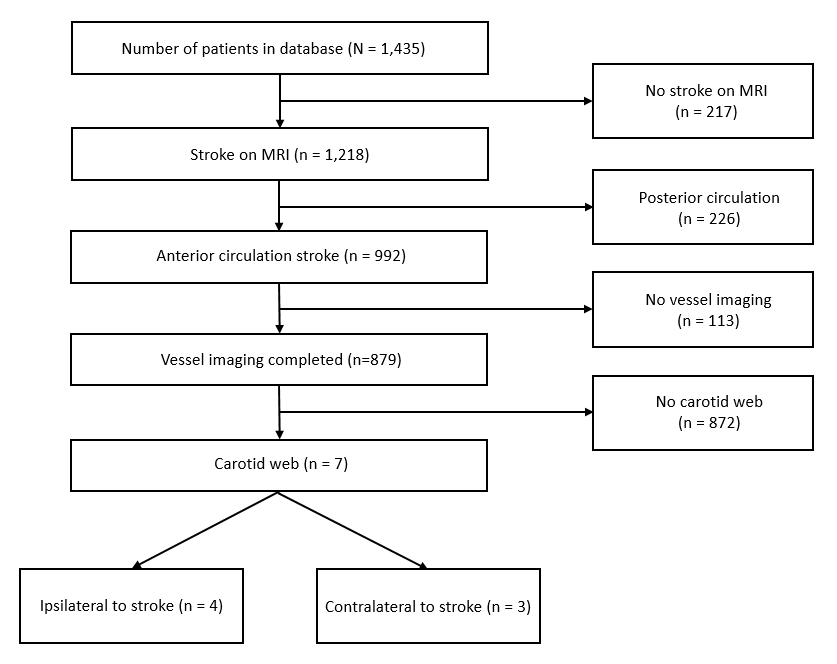


**S1 Fig.** **Flowsheet of patients included in our analysis.**

Supplement: S1 Fig — (DOCX) [file pone.0257697.s002.docx]

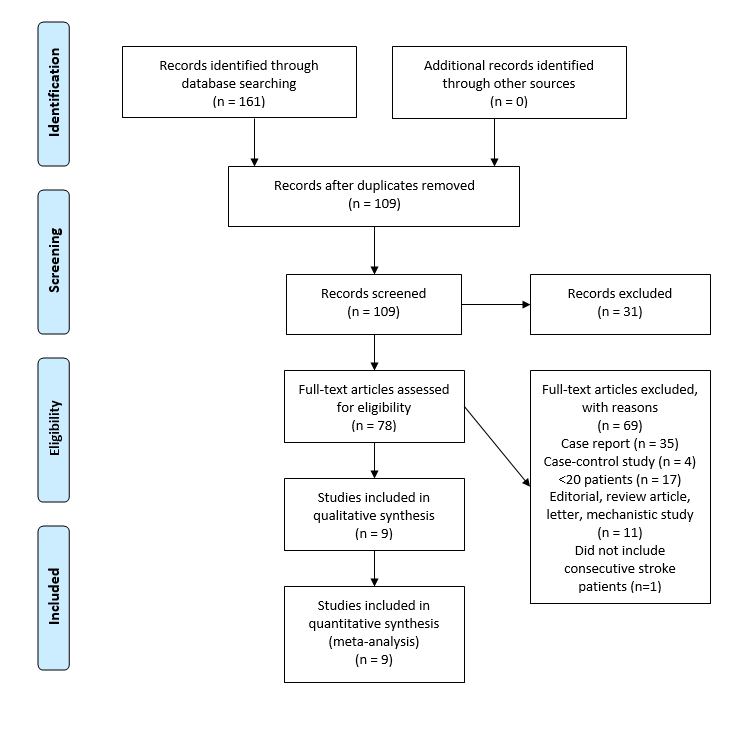


**S2 Fig.** **PRISMA Diagram of Study Selection.**

Supplement: S2 Fig — (DOCX) [file pone.0257697.s003.docx]
