# Supplementary material for: Ipsilateral internal carotid artery web and acute ischemic stroke: A cohort study, systematic review and meta-analysis
Source: PLoS One. 2021 Sep 17;16(9):e0257697. doi: 10.1371/journal.pone.0257697 (PMC8448368; doi:10.1371/journal.pone.0257697)
Supplement: S1 Methods — (DOCX) [file pone.0257697.s004.docx]

**Supplementary Methods**

**Systematic Review and Meta-analysis**

**Study Questions**

1. In people between the ages of 18 and 60 presenting with an acute cerebral ischemic stroke, what proportion of patients have a carotid web ipsilateral to the hemisphere with stroke?

2. What is the relative risk of carotid web ipsilateral versus contralateral to stroke? Does this differ when the question is restricted to patients with cryptogenic stroke, patients <60 or both?

**Inclusion criteria**

Adult (>18 years old) human patients.

Imaging documentation of acute anterior circulation cerebral ischemic stroke

Adjudication of presence/absence of carotid web

**Exclusion**

Cervical vessel imaging not performed.

Study includes less than 20 patients.

**Search strategy**

Search terms: “carotid web” or “carotid bulb web” OR “carotid bulb diaphragm”

Restrictions: Owing to the low anticipated number of studies, no restrictions were placed on article type, year of publication or language.

**Databases searched**

Pubmed, Web of Science, Cochrane Database of Systematic Reviews

**Screening process**

Studies were screened based on their title and abstract and - if deemed potentially relevant - the entire manuscript will be reviewed. We will extract the number of subjects in each study and the number of ipsilateral and contralateral carotid webs.

**Statistical analysis**

Prevalence of carotid web will be calculated for each study and pooled prevalence calculated via a random effects model. We will calculate relative risks of carotid web ipsilateral versus contralateral to stroke in the same pool of studies then do further analyses calculating this relative risk in patients with cryptogenic stroke then patients in cryptogenic stroke <60. Meta-analysis will be performed in R (R Foundation for Statistical Computing) using the R *meta* package.
