# Supplementary material for: Ipsilateral internal carotid artery web and acute ischemic stroke: A cohort study, systematic review and meta-analysis
Source: PLoS One. 2021 Sep 17;16(9):e0257697. doi: 10.1371/journal.pone.0257697 (PMC8448368; doi:10.1371/journal.pone.0257697)
Supplement: Attachment — Submitted filename: Response to Reviewers.docx [file pone.0257697.s006.docx]

**RESPONSE TO REVIEWERS & EDITORIAL BOARD**

We wish to thank the reviewers and the members of the editorial board for the time they have taken in the thoughtful review of our manuscript. We feel that our work has been greatly enriched by the incorporation of these comments. Please find our point-by-point responses below.

**Editorial Board:**

**1. Please ensure that your manuscript meets PLOS ONE's style requirements, including those for file naming.**

Thank you for this comment. We have updated the manuscript to adhere with PLOS ONE’s style requirements, including those regarding file naming.

The following changes have been made.

-Section headers have been changed to bold type, 18pt font.

-Section headers have been changed to sentence case.

-The style of figure callouts has been amended to comply with journal style.

-Figure captions have been added directly after the paragraph in which they are first cited.

-Figure titles now are included in bold type.

-Figures are uploaded separately as individual files.

-Subsections of major sections have been changed to bold type, 16 pt font.

-The table has been included directly after the paragraph in which it is first cited.

-References are cited in square brackets.

-Supplemental figures have been referenced as S1 Fig and S2 Fig.

-Acknowledgments section has been added.

-Supporting information captions are included at the end of the manuscript under a Level 1 heading.

-Supporting information files have been uploaded individually.

-The title and affiliations page has been modified to comply with journal style.

**2. We note that you have indicated that data from this study are available upon request. PLOS only allows data to be available upon request if there are legal or ethical restrictions on sharing data publicly… We will update your Data Availability statement on your behalf to reflect the information you provide.**

-Thank you. We apologize for this oversight. We have uploaded an accompanying file entitled “Anonymized data set” as supporting information. This has also been mentioned in the author cover letter.

**3. Please include captions for your Supporting Information files at the end of your manuscript, and update any in-text citations to match accordingly.**

-We have now included captions for our Supporting Information files at the end of the manuscript.

**Reviewer #1:**

**The manuscript by Mac Grory et al describes an observational study on patients with ischemic stroke and carotid webs as well as a systematic review and a meta-analysis. They found that an ipsilateral carotid web occurs in 0.45% of their cohort and in 4.5% in younger patients with a cryptogenic stroke. The pooled prevalence of an ipsilateral carotid web was 13%. The potential role of carotid webs in cryptogenic stroke is interesting. I have one comment:

The pooled prevalence of 13% of ipsilateral carotid web in younger patients with cryptogenic stroke seems rather high. Could this estimation be biased?**

-Thank you for this comment. This 13% estimate may certainly be a high estimate and may be in part mediated by publication bias. The confidence intervals are wide and there is a high degree of heterogeneity between studies. We have added the following limitation to the “Discussion” section:

“Our study benefited from a large cohort of patients from a comprehensive stroke center in whom stroke etiology was adjudicated by two separate investigators. Our findings should be considered in terms of a number of limitations: 1) we did not have pathological confirmation of intimal FMD, which is definitively diagnostic of carotid web(15); 2) within our meta-analysis, the estimated pooled prevalence of 13% of patients <60 with cryptogenic stroke may be an artificially high estimate in part mediated by publication bias 3) only one other study(16) in the systematic review classified stroke as ESUS and thus there is a small limitation introduced through combining studies with different definitions of cryptogenic stroke and; 4) because our study sample is not population-based, we cannot draw inferences on the prevalence of carotid web in the population at large.”

**Reviewer #2:**

**The present manuscript investigates the prevalence of carotid webs in stroke patients.
There are some ‘typos’ and inconsistencies in the text (for instance the phrase: Carotid web is an important possible stroke mechanism), please review for potential corrections.**

-Thank you very much for this comment. We have corrected the following typos and inconsistencies:

1. **“**In this study, we aim to determine the prevalence of ipsilateral carotid web~~s~~ in a cohort of ischemic stroke patients and to perform a systematic review and meta-analysis of similar cohorts.**”**

2. “Carotid web was defined on computed tomography angiography (CTA) as a thin shelf of non-calcified tissue immediately distal to the carotid bifurcation.”

3. “Carotid web is an important potential stoke mechanism…”

4. “The lower prevalence of carotid web in our study may ~~be a reflection of~~ reflect the…”

**Please also check and provide feedback for the following:
The study by Sajedy 2019 reports on the incidence of carotid web among consecutive CTA neck studies, not stroke patients, please explain how this may serve to calculate risk ratios for stroke.**

-The authors greatly appreciate this comment. As this was study was a study of consecutive CT angiograms of the neck and not of patients with stroke, its inclusion was erroneous. We have removed it from the current analysis and updated Figure 2A, the abstract, results and PRISMA diagram to reflect this. The removal of this study from the analysis of relative risk of ipsilateral versus contralateral carotid web from 2.6 (95% CI:1.6-4.3) to 2.5 (95% CI:1.5-4.2).

**Moreover, Ospel et al reports mainly on carotid plaques, not webs. Alternatively, you may remove both studies from the analysis.**
-Thank you for this comment. Although the study of Ospel et al was primarily concerned with non-stenotic carotid plaques, they did report on consecutive stroke patients from a prospective study with CTAs of the neck performed. They provided sufficient granularity in their reported results to allow us to determine the number of carotid webs. The numbers abstracted from this paper refer to carotid web specifically and not atherosclerotic plaques.

**The studies by Yo, and Labeyrie et al, which are used in the meta-analysis are not referenced at al. Does Mc Grory et al 2020, reported in the meta-analysis refer to the present study or just to the review referenced in the Supl?**

-Thank you very much for this comment. We have added in references to Yu et al. and Labeyrie et al. in the section under Results: Systematic review and meta-analysis. Mac Grory et al. refers to the present cohort and not the prior review paper. We have added the following clarification to the study legend:

“Please note that “Mac Grory 2021” refers to the present study.”
